# Successful treatment of blepharitis with bibrocathol (Posiformin^®^ 2 %)

**DOI:** 10.1007/s00417-012-2001-0

**Published:** 2012-04-25

**Authors:** Pavel A. Bezdetko, Nikolai Sergienko, Yuriy Dyomin, Andrei Korol, Nik Nikitin, Matthias Merzbacher, Dorothea Groß, Ralf Kohnen

**Affiliations:** 1 Department of Ophthalmology, Kharkov District Clinical Hospital, Kharkov, Ukraine; 2 Department of Ophthalmology, National Medical Academy of postgraduate Education, Kiev, Ukraine; 3 Post Diploma Education Academy, Kharkov, Ukraine; 4 The Filatov Institute of Eye Diseases and Tissue Therapy of AMS of Ukraine, Odessa, Ukraine; 5 RUSSLAN Clinical Research Ltd., North Humberside, UK; 6 RPS Research Germany GmbH, Nuremberg, Germany; 7 URSAPHARM Arzneimittel GmbH, Industriestraße 35, 66129 Saarbrücken, Germany; 8 ReSearch Pharmaceutical Services Inc, Fort Washington, PA USA; 9 Psychology Department, University of Erlangen-Nuremberg, Nuremberg, Germany

**Keywords:** Blepharitis, Bibrocathol, Treatment outcome

## Abstract

**Background:**

Bibrocathol is a well-established antiseptic drug for the treatment of acute eyelid diseases like blepharitis. Despite its frequent use in clinical practice, no controlled clinical trial on the efficacy of bibrocathol 2% eye ointment has been performed until now. The aim of the study was to investigate efficacy, safety and tolerability of bibrocathol (Posiformin® 2 %) eye ointment in patients diagnosed with blepharitis.

**Methods:**

In this multi-center, randomized, double-masked, placebo-controlled parallel-group comparison, the change of signs and symptoms (sum score) of blepharitis in 197 patients (ITT (intention-to-treat-group); mean age 56 ± 18 years, 56 % female, active drug:vehicle = 97:100) over 2 weeks treatment with bibrocathol 2 % eye ointment was evaluated.

**Results:**

Patients receiving bibrocathol 2 % showed greater improvement in the sum score than the placebo patients (*p* < 0.0001, Cohen’s effect size d = 0.73). Also, the results from further efficacy assessments improvement of single symptoms and ocular discomfort measured by a VAS (visual analogue scale) supported treatment with bibrocathol. Patients and investigators provided favorable tolerability ratings preferring bibrocathol over placebo. No safety issues were observed with regard to intraocular pressure, visual acuity, or occurrence of adverse events.

**Conclusions:**

Blepharitis therapy with the antiseptic bibrocathol 2 % in this trial was highly efficacious and safe.

## Introduction

Inflammation of the palpebra and the edge of the palpebra (blepharitis) is one of the most frequent diseases seen by the ophthalmologist [1, 2]. Predominant symptoms are burning, itching, and foreign-body sensation with swollen, hyperemic eyelids and debris.

Staphylococcal blepharitis, seborrheic blepharitis, and mixed forms are summarized as anterior blepharitis in contrary to posterior blepharitis, which is caused by a dysfunction of the Meibomian glands. Altered composition of secretions is frequently associated with specific clinical abnormalities such as evaporative dry eyes [2, 3].

Standard treatment of blepharitis is lid hygiene. According to the guidelines of the Professional Society of German Ophthalmologists and the German Ophthalmologic Association, lid hygiene should be supplemented by antibiotics or antiseptics, depending on disease cause and related findings [4, 5]. Topical antibiotics may be administered in case of severe infection, with additional topical steroids or steroid-antibiotic combination preparations if necessary [4–6].

Bibrocathol (International non-proprietary name (INN) 4,5,6,7-Tetrabromo-1,3,2-benzodioxabismol-2-ol) is a topical antiseptic frequently used for the treatment of blepharitis. Eye ointments containing 2 or 5 % bibrocathol and the excipients liquid paraffin, white soft paraffin, and lanolin have been marketed since 1967 for the treatment of eye irritation, chronic blepharitis, and uninfected corneal injuries. Reports of clinical experience with bibrocathol for inflammation of the edge of the palpebra exist since the beginning of the 20th century [7]. Until recently, no controlled, randomized clinical studies according to the guidelines for Good Clinical Practice for Trials on Medical Products for Human Use (GCP) as defined by the International Conference on Harmonisation (ICH) have been performed with bibrocathol 2 % ointment, as these were not required for marketing authorization in the 1960s. A first double-blind, prospective, controlled, GCP-compliant clinical study was recently performed to assess the efficacy of bibrocathol 5 % (Noviform®) in acute blepharitis [8]. It demonstrated superior efficacy of bibrocathol 5 % ointment as compared to an ointment vehicle (placebo) after 2 weeks of treatment as assessed by a combined measure of slit-lamp examination results and patients’ subjective complaints. However, the effect size in this study was small. Currently, only the 2 % bibrocathol eye ointment is available on the market (Posiformin® 2 %); however, no data from a modern clinical trial according to the standards of ICH-GCP exist to demonstrate its efficacy and tolerability.

This controlled multicenter clinical trial was performed to evaluate the efficacy and safety of bibrocathol 2 % eye ointment in the treatment of patients with acute blepharitis.

## Materials and methods

As all four participating trial sites were located in the Ukraine, this trial was approved by the Central Ethics Committee of Kyiv. It was conducted according to the guidelines for ICH - GCP and the Declaration of Helsinki, Tokyo 2004, as well as being in compliance with national Ukrainian law and regulations.

All patients provided written informed consent to voluntarily participate in the trial and to their personal data being inspected by employees of the sponsor, sponsor’s representatives, or authorities.

The study was designed as a multi-center, randomized, double-masked, placebo-controlled parallel-group trial evaluating the effects of bibrocathol 2 % eye ointment on acute blepharitis. Patients were randomly assigned in a 1:1 ratio to receive either bibrocathol 2 % or vehicle (placebo). The allocation schedule was generated by the Biometrics Department of ReSearch Pharmaceutical Services’ coding system and contained blocks of six randomization numbers—three assigned to each treatment arm in random order. Investigators were not aware of the block size used. The total treatment period was 14 (± 2) days with four visits at the investigational site (screening visit [V1], baseline visit on day 1 [V2], control visit [V3] on day 7 ± 1 and final visit [V4] at day 14 ± 2). A length of 5 mm bibrocathol 2 % or corresponding vehicle was applied to both the upper and lower eyelids up to the eyelid margin with a clean fingertip three times daily (morning, noon, and evening), after eyelid hygiene, starting on day 1 (V2). All patients were trained to apply the same measures of lid hygiene (washing the face and cleaning the lid with a cotton bud according to standards of each study center). Eye cosmetics and eyelid massage as well as wearing contact lenses were prohibited.

The identities of the treatments were concealed by study drugs that were identical in packaging, labeling, size, and appearance of tubes as well as the schedule of administration. Since active treatment and vehicle differed in color, the study medication was distributed to the patients by qualified site personnel not involved in the measurement of any trial efficacy or safety parameters. These personnel were instructed not to reveal the identity of the trial medication to the investigator or study monitors.

Male and female patients aged ≥ 18 years with signs and symptoms of moderate blepharitis (sum score of at least 15 out of 26 points) in ambulatory treatment were considered for participation. This sum score using a distinct grading of symptoms was developed in a previous study [6] and combined both the physicians’ evaluation of the four signs and symptoms of blepharitis severity (lid edema, lid erythema, debris and pouting of Meibomian glands, with a maximum of 16 points) and the patients’ subjective discomfort rating using a visual analogue scale (VAS) (maximum of 10 points).

Patients were excluded from the study if they suffered from blepharitis requiring antibiotic treatment or therapy-resistant blepharitis, had an abnormal eyelid anatomy (other than due to blepharitis), ocular surgery within the last 90 days, severe dry eye syndrome, an allergic eye disease, known hypersensitivity to the trial drug or any of the ingredients, severe systemic disease, rheumatoid arthritis or spondylitis, or a history of malignancies of any organ system within the last 5 years. The following concomitant medications led to exclusion from the trial: oral or topical antibiotics, topical ocular or systemic corticosteroids (except chronic use of inhaled corticosteroids on stable dose), topical ocular or systemic NSAIDs (except oral acetyl salicylic acid and occasional use of painkillers) administered 1 month prior to and during the trial, any other ocular antiseptics administered during the trial, local ocular use of antihistamines, and any ocular α-sympathomimetics. Eye cosmetics, eyelid massage, changes in lid hygiene regimen, and wearing contact lenses were prohibited during the trial. Pregnant or breast-feeding women were excluded from participation, as well as patients whose concurrent systemic therapies could affect the trial parameters, who participated in another clinical trial at the same time or were already included in the trial, and patients with any condition which in the investigator’s opinion would preclude the patient from adhering to the protocol requirements.

The primary objective of this trial was to demonstrate superiority of 2 weeks of treatment with bibrocathol 2 % eye ointment versus corresponding vehicle (placebo) in reducing signs and symptoms of blepharitis. The secondary objectives were to compare further measures of efficacy, tolerability, and safety of 2 weeks of treatment with bibrocathol ointment versus vehicle.

The primary efficacy parameter (assessed on V2, V3, V4) was the sum score of four signs and symptoms of blepharitis (severity of lid edema, lid erythema, debris, and pouting of Meibomian glands) according to slit-lamp examination of the worst affected eye at baseline (maximum 16 points in total from 0–4 points per sign/symptom; higher points indicate more severe signs/symptoms). The secondary efficacy parameters included the four single signs/symptoms of blepharitis separately (V2, V3, V4), antiseptic effect by palpebral smear taken from the study eyelid on a 4-point scale (higher points indicate more widespread growth of bacteria; assessed on V2, V4) and subjective ocular discomfort assessed by a visual analogue scale (VAS) ranging from 0 to 100 mm (V2, V3, V4). For the ocular discomfort assessment, the original ratings were transformed by mathematical rounding rules to a scale ranging from 0 to 10 with 10 reflecting maximum discomfort. The four grading stages mentioned above were clearly defined for each item and could easily be assessed by the participating physicians; they had been developed in a previous study [6]. Bacterial growth was assessed in local site laboratories by analysis of palpebral smear, taken from the study eye lid, with the following categories and scores: 0 = no growth of bacteria; 1 = scattered growth of bacteria (< 7 germs); 2 = moderate growth (7–50 germs) and 3 = widespread growth (> 50 germs).

Safety parameters consisted of a visual acuity test (performed on V3 and V4), intra-ocular pressure (IOP) measurement (V2, V4), change in concomitant ocular medication (V2, V3, V4), global tolerability assessed by the patient and the investigator on a 4-point scale with the highest figure indicating best tolerability (V4), adverse events (V3, V4), and vital signs examination (V3, V4).

Treatments were compared with respect to the efficacy variables in an analysis of covariance (ANCOVA) model with the factors of treatment and center. As baseline values were additionally considered to have an influence on changes from baseline, they were each included as covariates in the ANCOVA model. The treatment contrast was presented by a least squares (LS) mean for the group difference together with its 95 % confidence interval (CI) and the *p* value for the presumption that the contrast is 0. Effect sizes (Cohen’s d) were presented for group comparisons.

The absolute and relative frequencies as well as group differences by Chi-squared tests were calculated for categorical variables. The last observation carried forward method (LOCF) for substitution of missing data was used for an endpoint analysis in the primary and all secondary outcome measures. In the modified intent-to-treat (ITT) population, patients without post-baseline values for the primary efficacy variable were excluded from the analysis. For all other patients, missing values of signs/symptoms were replaced by the LOCF method (last observation carried forward). Other missing values were not replaced. Baseline characteristics were summarized descriptively for the ITT sample. Safety and tolerability variables were summarized descriptively for the safety sample.

The sample size estimation was based on a similar study with bibrocathol reporting an effect size of Cohen’s d = 0.43 for the difference to placebo with a slightly different primary efficacy variable [8]. Assuming that the primary efficacy measure was as sensitive as the outcome measure of the referenced trial, 86 patients per group were required to detect such an effect size with a power of 90 % at the two-sided significance level of 5 %. To account for non-evaluable data due to drop-outs, 2 × 100 = 200 patients were to be recruited for participation in this trial.

## Results

A total number of two hundred patients was enrolled in the trial, randomized, received bibrocathol 2 % (*n* = 100) or placebo (*n* = 100), and were available for safety and tolerability analysis. Three patients were excluded from the efficacy analysis as they discontinued the study prematurely without valid post-baseline data. Therefore, the ITT population (for demographic data see Table 1) consisted of 197 patients.

**Table 1:** Demographic data at screening (ITT)

Demographic variables	Statistic	Bibrocathol 2 %	Placebo	Total
		*n* = 100	*n* = 97	*n* = 197
Age (years)	M ± SD	57.3 ± 18.15	54.1 ± 17.90	55.7 ± 18.05
	Median	61.0	56.0	59.0
	Range	19–87	20–86	19–87
Sex				
Male	*n* (%)	42 (42.0)	45 (46.4)	87 (44.2)
Female	*n* (%)	58 (58.0)	52 (53.6)	110 (55.8)
Ethnicity				
Caucasian	*n* (%)	99 (99.0)	97 (100)	196 (99.5)
Asian	*n* (%)	1 (1.0)	--	1 (0.5)
BMI (kg/m^2^)	M ± SD	27.4 ± 3.58	26.6 ± 3.63	27.0 ± 3.61
	Median	27.7	26.4	27.1
	Range	16.6–37.1	17.5–35.4	16.6–37.1

*n* number of subjects, *%* percent of subjects in each group, *M* arithmetic mean, *SD* standard deviation, *range* minimum–maximum

### Efficacy

**Fig. 1:**
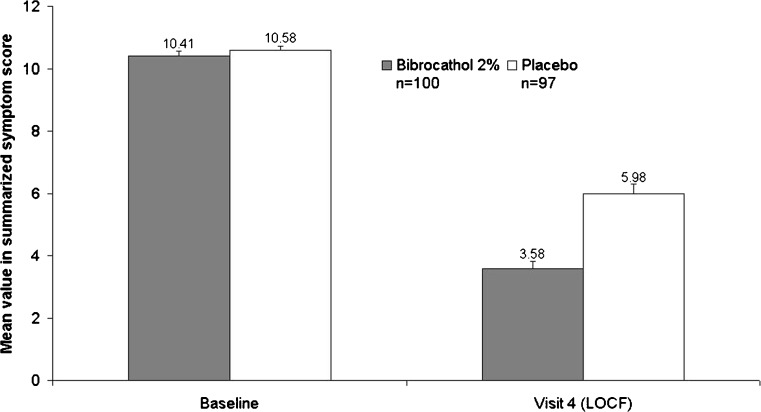
Sum score from slit-lamp examination (ITT)

Baseline mean values of the sum score were very similar in both groups (bibrocathol: 10.4 points, placebo: 10.6 points). At V4 (LOCF) in both groups, the score was reduced (raw values are given in Fig. 1); however, mean (± SD) reduction from baseline was larger in the group treated with bibrocathol (−6.8 ± 2.45 points) as compared to the placebo group (−4.6 ± 3.33 points). Difference between the treatment groups (LS mean) was −2.32 (95 % CI: –2.84; –1.80) favoring treatment with bibrocathol (*p* < 0.0001 from ANCOVA; Cohen’s d = 0.73).

**Fig. 2:**
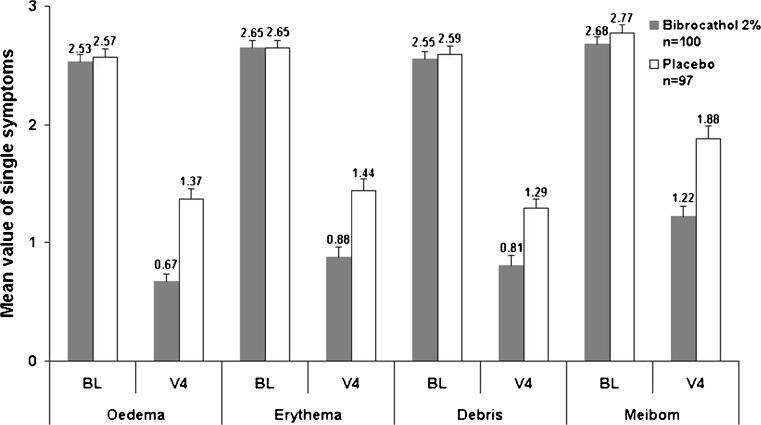
Single scores from slit-lamp examination (ITT)

Baseline means in the four sub-scores were almost equal between the two groups. Until V4 all signs/symptoms improved to a larger extent under bibrocathol than under placebo (for raw values see Fig. 2; differences are given in Table 2).

**Table 2:** Secondary efficacy variables; results from ANCOVA (ITT)

Change from BL (LOCF)	Statistic	Bibrocathol 2 %	Placebo
		*n* = 100	*n* = 97
Edema	M ± SD	−1.86 ± 0.89	−1.20 ± 1.20
	Median (range)	−2.00 (−4–1)	−1.00 (−4–1)
Difference	LS mean (95 % CI)	−0.70 (−0.88; –0.51)	
	*p* value	<0.0001	
	Cohen’s d	0.60	
Erythema	M ± SD	−1.77 ± 0.85	−1.21 ± 1.15
	Median (range)	−2.00 (−4–0)	−1.00 (−4–0)
Difference	LS mean (95 % CI)	−0.57 (−0.75; –0.38)	
	*p* value	<0.0001	
	Cohen’s d	0.54	
Debris	M ± SD	−1.74 ± 0.89	−1.30 ± 0.82
	Median (range)	−2.00 (−4–0)	−1.00 (−4–0)
Difference	LS mean (95 % CI)	−0.47 (−0.65; –0.29)	
	*p* value	<0.0001	
	Cohen’s d	0.50	
Pouting of Meibomian glands	M ± SD	−1.46 ± 0.80	−0.90 ± 1.08
	Median (range)	−1.00 (−4–0)	−1.00 (−4–0)
Difference	LS mean (95 % CI)	−0.62 (−0.82; –0.43)	
	*p* value	<0.0001	
	Cohen’s d	0.57	
VAS (0–10)	M ± SD	−4.69 ± 1.95	−2.79 ± 2.29
	Median (range)	−5.00 (−10–1)	−2.00 (−9–1)
Difference	LS mean (95 % CI)	−1.86 (−2.25; –1.46)	
	*p* value	<0.0001	
	Cohen’s d	0.81	

*n* number of subjects, *%* percent of subjects in each group, *M* arithmetic mean, *SD* standard deviation, *range* minimum–maximum, *p* values result from ANCOVA

**Fig. 3:**
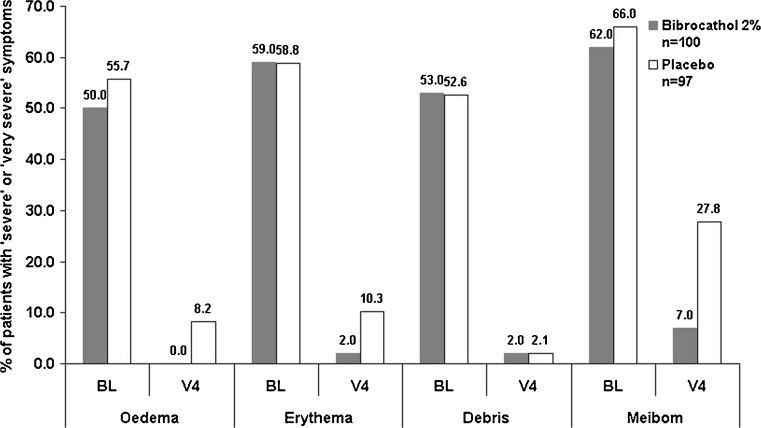
Frequency distribution of patients with ‘severe’ or ‘very severe’ symptoms at baseline and last visit (LOCF) [ITT]

Almost all patients had at least one ‘severe’ or ‘very severe’ symptom in the blepharitis slit-lamp examination at baseline (bibrocathol: 97.0 %, placebo: 97.9 %). At V4 (LOCF), only 11 patients (11.0 %) in the bibrocathol group suffered from one such symptom. In the placebo group, still 31 (32.0 %) patients were affected with 12 patients having more than one ‘severe’ or ‘very severe’ symptom (see Fig. 3 for frequency distribution). The group difference at V4 (LOCF) was statistically significant with *p* < 0.001 in all four signs/symptoms (Chi-square test).

The patients of both groups rated their subjective ocular discomfort to be about 7.4 points on average at baseline (10 = very severe). In the bibrocathol group, ocular discomfort improved significantly more than in the placebo group (see Table 2). Scattered (< 7 bacteria) or moderate (8–50 bacteria) bacterial growth could be revealed at baseline in 53 % of the bibrocathol group and in 53.6 % of the placebo group. Bacteria could no longer be identified at study end in 35.8 % of these patients under bibrocathol and in 32.7 % under placebo.

### Safety/tolerability

Visual acuity changed marginally between baseline and V4 on average (bibrocathol: 0.00 ± 0.04 %; placebo: 0.01 ± 0.09 %). Only five patients (four bibrocathol, one placebo patient) reported very small changes of vision (%). Mean IOP was slightly reduced between baseline and V4 in both groups (bibrocathol: –0.45 ± 1.66; placebo: –0.34 ± 1.78). Tolerability of bibrocathol was reported to be ‘good’ or even ‘very good’ by almost all patients (93.0 %). Investigators’ ratings were even more favorable for bibrocathol, with 95.0 % patients classified as showing at least ‘good’ tolerability of bibrocathol. In the placebo group, only 47.0 % of the patients’ and 35.0 % of the investigators’ ratings reflected at least ‘good’ tolerability.

No patient took other ophthalmological medication than the study medication prior to or during the study. No relevant changes with regard to vital signs occurred.

Adverse events (AEs) were rare in the bibrocathol group with six (6.0 %) patients affected by administrative site conditions (application site pruritus in one patient and application site reactions in the remaining five), which were considered to be related to the study medication. The investigators reported AEs for 66 (66.0 %) placebo patients: two patients experienced hypersensitivity against the ointment, 28 experienced application site pruritus, and 36 reported application site reactions. Almost all AEs were mild and lasted only for a few minutes; only two patients discontinued the trial due to AEs.

## Discussion

The results of this study clearly demonstrate that bibrocathol 2 % ointment was superior over vehicle ointment (placebo) with regard to improvement of blepharitis symptoms after 2 weeks of treatment in a patient population suffering from acute palpebral inflammation but not requiring antibiotic treatment. As infection plays a crucial role in the etiology of blepharitis, adding antiseptic agents to standard lid hygiene seems to be a reasonable and effective treatment option, especially as it avoids the risk of bacterial resistance and substance hypersensitivity associated with topical use of antibiotics, at the same time decreasing the risk of side-effects of antibiotics [1, 2, 9, 10]. Bibrocathol 2 % is an antiseptic ointment with disinfectant, anti-inflammatory, astringent, and secretion-inhibitory effects. Its mechanism of action is determined by its structural components, mainly tetrabromopyrocatechol and bismuth hydroxide. Bacterial invasion is prevented by protein denaturation and surface tissue diminution. In addition, the astringent effect on small vessels reduces local inflammation and secretion [11]. Therefore, no resistance to this antiseptic agent can develop.

This mechanism of action is different from the antibacterial effect of antibiotics. Previous in-vitro microbiological tests had shown a potential of an antibacterial effect (data on file). To confirm this property in a clinical setting, palpebral smears were taken during this study. However, these tests could not show a relevant difference between active treatment and placebo. It was concluded from these tests that the clinical mode of action of bibrocathol 2 % is not a direct antibacterial effect comparable to antibiotic treatment. However, further evidence is needed to support the pharmacodynamic effect of bibrocathol.

The observed treatment differences between bibrocathol 2 % and placebo are clinically relevant in the total population and also in severely affected patients. This is indicated on the one hand by the significant treatment differences regarding the total score and the sub-scores with medium to large effect sizes. In clinical trials, medium-effect sizes (d = ~0.5) are usually considered the lower limit of clinical relevance. In our study, the effect size was 0.73 for the primary endpoint, which is clearly above the ‘medium’ level and close to the requirement for a ‘large’ effect (d ≥ 0.8). Thus, the superiority of bibrocathol 2 % over placebo is not only statistically significant but also clinically relevant. On the other hand, there were very few patients with severe or very severe symptoms in the bibrocathol group as compared to the placebo group although vehicle therapy also showed a curing effect at the end of the 14-day treatment period. Additionally, by defining a responder as a patient with a total score <4 and each of the 4 signs/symptoms being ≤1, we found 58.0 % responders in the bibrocathol and 25.8 % responders in the placebo group (*p* < 0.0001 in Fisher’s exact test).

The high number of application site reactions or pruritus in the placebo group may rather be attributable to disease symptoms, which did not improve under therapy than to AEs caused by the placebo ointment, as their occurrence was associated with a higher blepharitis total score in both groups (correlation of ρ = 0.69 for the total sample). This is in accordance with our expectation that the application of eye ointment could possibly cause application site discomfort, which may be perceived more intensely by patients with no or slow symptom improvement than by patients with fast symptom improvement.

Taking the favorable tolerability and safety of bibrocathol 2 % eye ointment into account, together with the clinically relevant efficacy as shown by symptom improvement, it is concluded that the antiseptic bibrocathol 2 % is an efficacious therapy of acute forms of blepharitis with a positive risk–benefit ratio. In clinical practice, it can help to avoid the risks of antibiotic treatment including bacterial resistance or hypersensitivity reactions. Further clinical investigations may include a long-term follow-up over several months, and other trials could prove the efficacy of bibrocathol 2 % in long-term treatment to confirm its benefits in chronic forms of blepharitis.
